# Identification of subspecies-divergent genetic loci responsible for mineral accumulation in rice grains

**DOI:** 10.3389/fgene.2023.1133600

**Published:** 2023-02-07

**Authors:** Zijian Huang, Sai Li, Zhaokun Lv, Yan Tian, Yibo Chen, Yuxing Zhu, Jiurong Wang, Huabing Deng, Liang Sun, Wenbang Tang

**Affiliations:** ^1^ College of Agronomy, Hunan Agricultural University, Changsha, China; ^2^ Key Laboratory of Agro-Ecological Processes in Subtropical Region, Institute of Subtropical Agriculture, Chinese Academy of Sciences, Changsha, China; ^3^ State Key Laboratory of Hybrid Rice, Hunan Hybrid Rice Research Center, Changsha, China

**Keywords:** mineral accumulation, rice, phenotypic normalization, QTL, subspecies differentiation

## Abstract

Rice (*Oryza sativa* L.) is a major staple food that provides not only dietary calories but also trace elements for the global inhabitants. The insufficiency of mineral nutrients and the potential accumulation of excessive toxic elements in grains pose risks to human health. The substantial natural variations in mineral accumulation in rice grains presents potentials for genetic improvements of rice *via* biofortifications of essential mineral nutrients and eliminations of toxic elements in grains. However, the genetic mechanisms underlying the natural variations in mineral accumulation have not been fully explored to date owing to unstable phenotypic variations, which are attributed to poor genetic performance and strong environmental effects. In this study, we first compared the genetic performance of different normalization approaches in determining the grain-Cd, grain-Mn, and grain-Zn variations in rice in different genetic populations. Then through quantitative trait loci (QTLs) identification in two rice inter-ectype populations, three QTLs, including *qCd7*, *qMn3*, and *qZn7*, were identified and the QTLs were found to exhibit allelic differentiation in the different ecotypes. Our results were expected to broaden our understanding for mineral accumulation in rice and propose the potential functional alleles that can be explored for further genetic improvement of rice.

## Introduction

As a major staple crop, rice (*Oryza sativa*) feeds nearly half of the human population worldwide. Rice and its derivates serve as the main source of calories, especially for the inhabitants of Southeast Asia (Miura et al., 2011). Rice is also the major dietary source of essential minerals and toxic elements in these populations (White and Broadley, 2009; Clemens and Ma, 2016). The essential mineral nutrients, including Fe and Zn, are often insufficient in rice (Chaney, 2015). The average concentrations of Fe and Zn in the current varieties of polished rice are 2.0 and 16.1 mg/kg, respectively, which are way lower than the recommended levels of 15 and 28 mg/kg for Fe and Zn, respectively (Bouis et al., 2011; Bashir et al., 2013). Over 60% and 30% of the global population feeding on rice suffer from Fe and Zn deficiency, respectively (White and Broadley, 2009). This scenario for malnutrition of essential mineral nutrients, known as “hidden hunger”, has become a worldwide concern for people feeding on rice (Slamet-Loedin et al., 2015). Additionally, rice grains accumulate high levels of toxic heavy metal elements, including Cd and As, which leads to a relatively high risk of intake of excessive toxic elements (Clemens and Ma, 2016; Zhao et al., 2022). Evidences that rice is the major contributor to dietary Cd exposure worldwide have been manifested (UNEP, 2008). High consumptions of rice in east Asia and Southeast Asia accounts for 90%, 31%, 46%, and 55.8% of dietary Cd in Vietnam, South Korea, Japan, and China, respectively (Song et al., 2017; Kuramata et al., 2022). The low levels of Fe and Zn in rice grains further escalate the risk of Cd intake in the human body (Chaney, 2015; Huang et al., 2021). The deficiency of essential minerals and enrichment of toxic heavy metals in rice grain is therefore a critical concern. There are numerous strategies for mitigating these risks, of which the genetic improvement of rice varieties is a fundamental and cost-effective approach for the biofortification of essential mineral nutrients and lowering the accumulation of toxic elements in rice grains (White and Broadley, 2009; Zhao et al., 2015).

There are substantial natural variations in mineral accumulation in the grains of different varieties or subspecies of rice. Previous reports have demonstrated that the concentration of minerals in rice grains vary from several fold to a few dozen fold among different varieties of rice, which poses potentials for exploring the genetic controls and genetic improvements on mineral accumulation in rice grains (Zhang et al., 2014; Yang et al., 2018; Tan et al., 2020a). The genetic mechanisms underlying the differences in mineral accumulation in rice grains have been previously explored by linkage or associated mapping studies, and multiple quantitative trait loci (QTLs) have been identified (Zhang et al., 2014; Yang et al., 2018; Yang et al., 2018; Wang et al., 2019; Wang et al., 2019; Tan et al., 2020a; Tan et al., 2020a). However, the majority of QTLs identified so far are minor QTLs and case-sensitive. Only a few QTL-genes (QTGs) have been functionally characterized, including *OsHMA4*, *OsHMA3*, and *OsCF1* (Ueno et al., 2010; Huang et al., 2016; Li et al., 2022). In additions, significant differences in the mineral accumulations in grains between rice have been documented between rice subspecies or among ecotypes [five ecotypes, as tropical *japonica* (*trj*), temperate *japonica* (te*j*), *aro*, *aus*, and *ind* ecotype] (Yang et al., 2018; Tan et al., 2020a). For instance, it has been reported that the *japonica* rice often accumulates more Mn, Zn, and Cu but less Cd in the grains compared to the *indica* rice, and the accumulation of Cd and As is often lowest in the *trj* ecotype among the five ecotypes (Sun et al., 2016; Tan et al., 2020a; Ruang-Areerate et al., 2021). At the molecular genetic levels, a limited number of functionally characterized genes, such as *OsHMA3*, *OsCd1*, *OsCF1*, and *OsLCT1* display functional subspecies divergence with amino acid polymorphisms in the protein sequences and/or differences in gene expression patterns/profiles (Ueno et al., 2010; Uraguchi et al., 2011; 2014; Zhou et al., 2017; Sui et al., 2019; Li et al., 2022). Beyond those, the genetic mechanisms underlying the differences of mineral accumulated in rice grains between subspecies or among ecotypes remain to be fully elucidated.

Environmental factors play great influence on driving the performance of mineral accumulation in rice grains (Zhang et al., 2014; Zhao et al., 2015; Tan et al., 2020a). Even if the heritability of some mineral accumulations in rice grain are relatively high, for examples, the broad heritability (*h* ^2^) of Cd, Cu, Mn, and Zn range from 0.39 to 0.87 (Yao et al., 2015; Yang et al., 2018; Tan et al., 2020a), the performance of mineral accumulation in rice grain are often determined largely by environmental changes (Zhao et al., 2015; Sun et al., 2022). Consequently, it is difficult to evaluate the genetic contributions to mineral accumulation in rice grain among difference rice varieties/lines, which further limits next genetic explorations. In order to address this issue, phenotype normalization approaches have been established for assessing the variations in genetic contributions to mineral accumulation in rice grains (Wang et al., 2019; Tan et al., 2020a; Sun et al., 2022). Based on the minimized effect of environmental heterogeneity in contrast-design field experiments (Lin and Poushinsky, 1983), we have optimized the experimental design and established a novel method of testing grain-Cd differences among different rice varieties/lines based on the enhanced genetic contributions to grain-Cd performance using the comparative measurement of grain-Cd accumulations between the test varieties/lines of rice and the control (Sun et al., 2022). While this method can successfully evaluate the grain-Cd performance, its applicability to the measurement of other minerals remains to be determined. On the statistics analysis levels, the Z-score, also known as standard score, is a useful statistical measure that has been successfully adopted for studying the QTLs responsible for mineral accumulation (Wang et al., 2019). Although both the normalization approaches have been proved to be successful in enhancing the genetic effects and limiting the effects of environmental changes on mineral accumulations (Wang et al., 2019; Sun et al., 2022), it is inquisitive to determine whether the genetic contributions can be further improved by combining the comparative mineral accumulation method with the Z-score.

In this study, we initially carried out phenotypic analysis of a recombinant inbred line (RIL) population to compare the potential of different phenotype normalization approaches in improving the genetic contribution to the variations in the performance of grain-Cd, grain-Mn, and grain-Zn in field experiments. Subsequently, QTL analysis was performed using the comparative mineral accumulation approach for evaluating the comparative grain-Cd (CCd), comparative grain-Mn (CMn), and comparative grain-Zn (CZn) accumulation in the two RIL populations derived from the progenies of crosses between a common *trj* variety and two *ind* cultivars to explore the genetic variations in grain mineral accumulation between different ecotypes. This study not only find a way to normalize phenotypic variations in mineral accumulation variations resulting from environment heterogeneity, but also offered potential QTG for isolating from the *trj* gene pools which could be used in the genetic improvement of rice *via* the biofortification of Mn and Zn and restricting Cd accumulation in rice grains.

## Materials and methods

### Plant materials and growth conditions

Five ecotypes of rice were categorized in this study, including *ind*, *aus*, *tej*, *trj*, and *aro,* based on the classification of rice ecotypes reported in the classical study (Garris et al., 2005). The differences in mineral accumulation in the grains of the five ecotypes were also determined in our previous studies (Sun et al., 2016; Tan et al., 2020a). Based on the performance of mineral accumulation, the “93–11” and “Teqing” varieties of *ind* rice were crossed with the “IRAT129” variety of *trj* rice for developing two biparental populations. Pop6-RIL was derived by intercrossing the “IRAT129” and “93–11” varieties, and Pop15-RIL was derived by intercrossing the “IRAT129” and “Teqing” varieties. The Pop6-RIL-F13 and Pop15-RIL-F11 varieties, consisting of 114 and 159 lines, respectively, were developed by the successive selfing of generations from the F1 descendant using the single seed descent method.

In order to investigate the accumulation of Cd, Mn, and Zn in the rice grains, the two genetic populations (Pop6-RIL and Pop15-RIL) were planted in our testing Cd contaminated paddy field. The levels of Cd in the soil ranged from 0.8 to 1.0 mg/kg and the pH ranged from 5.4 to 6.1, indicating that the soil was acidic. Based on our previously reported CCd evaluation approach (Sun et al., 2022), following the contrast design of adjusting the environmental heterogeneity (Lin and Poushinsky, 1983; Schaalje et al., 1987), the *ind* rice “93–11”, also being one of the parental varieties of RIL-Pop6, was employed to be planted adjoin to each line of Pop6-RIL in the paddy field for comparative mineral accumulation evaluations. Similarly, one parental line of RIL-Pop15, the *ind* rice “Teqing” was planted adjoin to each line of Pop15-RIL for comparative mineral accumulation evaluations. The two RILs were planted in two field conditions with two independent biological replications in each field (Supplementary Table S1). The field management essentially followed the local agricultural practices, except that the irrigation regimes. The solid compound fertilizer (N:P:K = 15:15:15) was applied at a concentration ∼750 kg/ha during the planting season. The intermittent irrigation management was adopted to keep water-holding capacity in the field between 60% and 100% in the field during the heading and filling stages for maximizing the phytoavailability of Cd in the soil.

### Construction of linkage genetic maps

In order to construct a linkage genetic maps of Pop6-RIL, each line of RIL was genotyped to build the genetic linkage map. For Pop6-RIL, a total of 237 genomic sequence tagged site (STS) markers and 71 simple sequence repeat (SSR) primers were randomly selected and developed for determining the genotypic polymorphisms of the molecular markers between the “IRAT129” and “93–11” parental varieties. All STS markers were developed and labeled in a previous study (Chen et al., 2021; Li et al., 2022) and all the SSRs primers were developed based on the GRAMENE database (http://www.gramene.org/). For Pop15-RIL, total 484 genomic markers, including 194 STS markers developed using the Primer design tool in RiceVarMap 2.0 (http://ricevarmap.ncpgr.cn) and 290 STS markers identified in our previous studies (Chen et al., 2021; Li et al., 2022), were developed for detecting the genetic polymorphisms of the molecular markers between the “IRAT129’” and “Teqing” parental varieties. The polymorphic markers in the different lines of the two RIL populations were genotyped using the SSR assay as previously described, with minor modifications (Zhou et al., 2019). After discarding the markers being indistinct or displaying distinct segregation distortion, the information of the available markers in each RIL line were genotyped. The genetic maps of Pop6-RIL and Pop15-RIL were subsequently constructed in computer program Mapmaker/Exp Ver3.0 (Lincoln et al., 1992). The linkage group were created with a minimum LOD threshold of 3.0 and the genetic distance between two markers calculated with the using Kosambi function. The genetic maps of Pop6-RIL and Pop15-RIL were plotted using Mapchart, version 2.32 (Voorrips, 2002; Supplementary Figure S1).

### Determination of grain-Cd, grain-Mn, and grain-Zn levels in the two RILs

Rice grains from each line of the two RILs were harvested and adequately air-dried for at least 2 weeks. After milling, the samples of brown rice from each line were subsequently oven-dried at 80°C to a constant weight for determining the concentrations of minerals, including Cd, Mn, and Zn, in the grains. The concentrations of minerals in the grains were determined as previously described (Zhou et al., 2019). The certified standard sample and the blank samples were similarly processed for assessing the accuracy of determination of the mineral concentrations. Then the concentration of minerals in the digested samples with duplications were detected in duplicate using an inductively coupled plasma emission spectrometer (Agilent Technologies 720 Series ICP-OES; Agilent, United States).

### Normalization of the levels of mineral accumulation in the RIL grains

The phenotypic performance of mineral accumulations in the rice grains were normalized by calculating two indicators, namely, the comparative mineral accumulation levels and the Z-score values, following their instructions (Wang et al., 2019; Sun et al., 2022). After determinations of the mineral accumulation in rice grain on the lines from each RIL and its corresponding control lines (“93–11” as control in Pop6-RIL, “Teqing” as control in Pop15-RIL), the comparative mineral accumulation levels of each tested line were evaluated. The CCd, CMn, and CZn of each tested RIL were calculated following the ratio of grain-Cd, grain-Mn, and grain-Zn levels between the tested line and its adjacent control line. For determinations of the Z-score values of grain-Cd, grain-Mn, and grain-Zn accumulation, denoted as CdZ, MnZ, and ZnZ, respectively, were determined using the formula: Z = (X-μ)/σ, where X represents the grain mineral accumulation in each line of the RIL, *µ* indicates the mean value of grain mineral accumulation in the entire RIL population, and *σ* represents the standard deviation of the grain mineral accumulation in the entire RIL population.

### QTL detection and statistical analysis

Using the mean values of each of the normalization approaches, QTLs detections were implemented for determining the additive QTLs using the inclusive composite interval mapping (ICIM) approach with the QTL IciMapping program, version 4.0 (Wang, 2009). The window in each run was <1.0 cM, and the *p*-value for the stepwise regression-based likelihood ratio test was set at the highest value of 0.05. The chromosomal regions containing markers with logarithm of odds (LOD) scores were determined using permutation test with 1,000 runs. The LOD value of 3.04 in Pop6-RIL and 3.41 on Pop15-RIL were eventually evaluated and regarded as significant additive QTLs. In order to compare the differences in QTL detection among the comparative mineral accumulation approach, Z-scores of grain-Cd levels, and the CZ values, QTL detections were also performed using the phenotypic data obtained from different normalization approaches for describing the differences among the three normalization approaches. The other statistical analyses, including correlation analysis and Student’s t-tests, were performed with Microsoft Excel 2019 (Microsoft Corporation) or SPSS 18.0 (SPSS Inc. PASW Statistics for Windows). In two-way ANOVA, total phenotypic variance was disintegrated into the variances of planting environmental changes, genetic variation, and their interactions between them. Then, following the fixed model, the expected variances (δ^2^) of environmental changes, genetic variation, and their interactions were also evaluated, then the proportion of each expected variance (δ^2^) in the total phenotype variance was estimated to describe the phenotypic contribution (Con%).

### Analysis of ecotypic differentiation of *qCd7*, *qMn3*, and *qZn7*

The QTL analysis for the two RIL populations jointly revealed three QTLs, namely *qCd7*, *qMn3*, and *qZn7*, being detected in both the RILs. The ecotypic diversity resulting from the three QTL regions was determined using a collection of 203-variety rice collection in the RiceVarMap database, version 2.0 (Zhao et al., 2021). The single nucleotide polymorphisms (SNPs) in the QTL regions of the 203 varieties of rice were randomly extracted. In detail, a total of 122, 115, and 134 SNPs was extracted from the genomic regions of *qCd7* (∼3.43 Mb), *qMn3* (∼4.6 Mb), and *qZn7* (∼3.98 Mb), respectively. The population genetic architecture of the 203 varieties in the collection was determined using the STRUCTURE software, version 2.3.4, with the maximum likelihood method under a burn-in of 10,000, a run length of 100,000 with three independent runs each, and a model allowing for admixture and correlated allele frequencies (Pritchard et al., 2000). The three independent runs yielded consistent likelihoods of the population structure for each predicted Q value. The subspecies and ecotypic variation of each QTL region was determined based on the calculated Q values and the data for each variety of rice.

## Results

### Extending the CCd method for comparative evaluation of grain-Mn and grain-Zn

In our previous study, the CCd evaluation approach was established based on the optimized experimental design. It was confirmed that the CCd approach excels in stably determining the grain-Cd among the different rice varieties and lines (Sun et al., 2022). In this study, we extended the CCd method for evaluating the genetic performance of other mineral elements (grain-Mn and grain-Zn) in rice grains. Z-score calculation is a powerful statistics algorithm for standardizing the performance of obtained data, and this strategy was also adopted for the phenotypic investigation of mineral accumulation in rice grains (Wang et al., 2019). We therefore determined if the combination of the Z-score with the CCd evaluation could further improve the genetic performance of mineral accumulation in rice grains. To this end, Pop6-RIL was planted in two different paddy fields, and the variations in grain-Cd, grain-Mn, and grain-Zn levels in the population were determined. The comparative mineral accumulation levels, Z-scores for mineral accumulation, and the CZ scores were determined for each line of the RIL populations according to the protocols for the normalization approaches.

**FIGURE 1:**
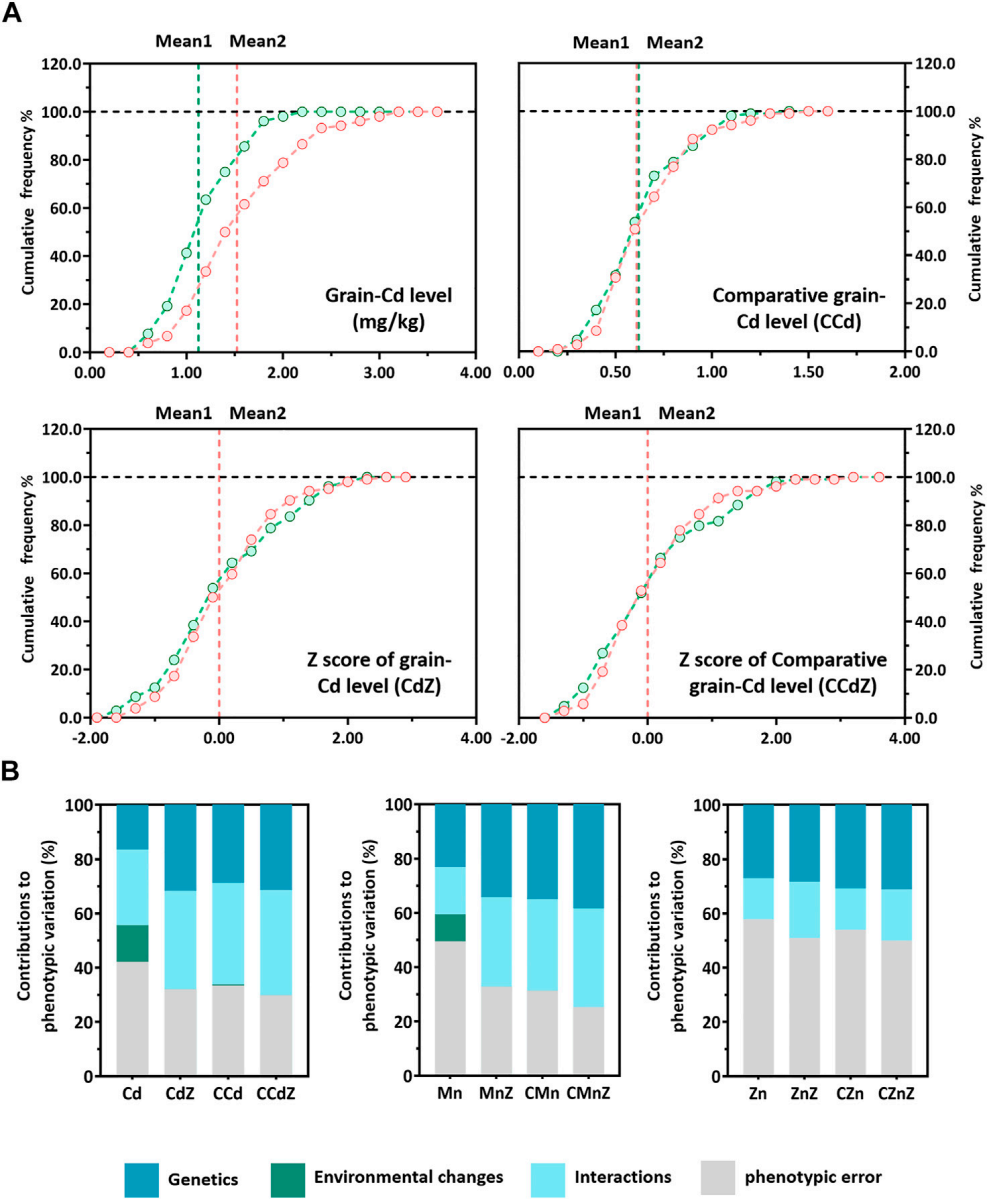
Performance of grain-Cd in the different normalization approaches and the different phenotypic components. **(A)** Performance of Cd accumulation in rice grains using different normalization approaches. **(B)** Contributions of different phenotypic components to the phenotypic variations in the different normalization approaches. The regions in blue, green, cyan, and gray represent the genetic contributions, environmental changes, interactions between genetic contributions and environmental changes, and the phenotypic error, respectively.

The genetic performance in the three phenotype normalization approaches was determined from the phenotypic distributions in the populations and phenotypic determinants revealed by two-way analysis of variance (ANOVA) of the phenotypic contributions (Table 1; Supplementary Figure S2). Analysis of the grain-Cd accumulation based on the CCd, CdZ, and CCdZ revealed that the fluctuations in the phenotypic distributions in the Pop6-RIL population were much lower than the variations in the grain-Cd levels, irrespective of the normalization approach used (Figure 1A). Analysis of the performance of grain-Cd based on the levels of CCd, CdZ, and CCdZ revealed that the performance of grain-Cd was significantly determined by the genotypic diversity between lines and the interactions between genotypic diversity and environmental changes (*p* < 0.01), while the environmental changes did not have a significant effect on the phenotypic variations (*p* > 0.05; Table 1). The findings suggested that the phenotype normalization approaches could eliminate the influence resulted from the environmental changes on the phenotypic performance. The genetic contributions to phenotypic variations enhanced from 13.55% in the grain-Cd level analysis to 28.85%, 31.79%, and 31.51% in CCd, CdZ, and CCdZ evaluations, respectively (Figure 1B; Table 1). Similarly, for grain-Mn accumulation, the environmental changes did not play a significant role in the variations in CMn or CMnZ variation either (*p* > 0.05; Table 1). The genetic contributions to the phenotypic variations were boosted from 9.96% in grain-Mn levels to 33.74%, 33.03%, and 36.29% in the CMn, MnZ, or CMnZ levels, respectively (Table 1; Figure 1B). However, the phenotypic normalization method proved less efficacious in increasing the genetic contributions that determined the variations in grain-Zn variation. The genetic effects of grain-Zn levels, CZn, ZnZ, and CZnZ exhibited slight variations, and were 15.05%, 15.24%, 20.71%, and 18.89%, respectively (Table 1). The findings revealed that phenotype normalization approaches can be successfully applied for determining the accumulation of grain-Cd and grain-Mn accumulation in rice, but invalid in determining the accumulation of grain-Zn accumulation in rice.

**TABLE 1:** The phenotypic components analysis using two-way ANOVA for grain-Cd, grain-Mn, and grain-Zn performance in Pop6 grown in two Cd-contaminated paddy fields.

Traits	Normalization approach	Variation components^a^	Sum square	df	Mean square	*F* value^b^	σ^2^	Con%^c^
Cd	None	Environ	16.88	1	16.88	67.85**	0.08	13.55
		Genotype	65.72	103	0.64	2.56**	0.09	16.49
		G × E	33.82	103	0.33	1.32*	0.16	27.81
		Error	51.76	208	0.25		0.25	42.15
	CCd	Environ	0.11	1	0.11	3.45	—	—
		Genotype	14.99	103	0.15	4.43**	0.03	28.85
		G × E	7.56	103	0.07	2.24**	0.04	37.56
		Error	6.83	208	0.03		0.03	33.59
	CdZ	Environ	1.14E-13	1	1.14E-13	2.6E-13	—	—
		Genotype	223.57	103	2.17	4.97**	0.43	31.79
		G × E	101.62	103	0.99	2.26**	0.49	36.18
		error	90.81	208	0.44		0.44	32.02
	CCdZ	Environ	1.14E-13	1	1.14E-13	2.77E-13	—	—
		Genotype	221.02	103	2.15	5.22**	0.43	31.51
		G × E	109.5	103	1.06	2.59**	0.53	38.62
		error	85.48	208	0.41		0.41	29.86
Zn	None	Environ	0.17	1	0.17	0.01	—	—
		Genotype	5278.79	94	56.16	2.04**	7.16	15.05
		G × E	2417.53	94	25.72	0.93	—	—
		Error	5230.84	190	27.53		27.53	57.9
	CZn	Environ	0.11	1	0.11	2.41	—	—
		Genotype	8.77	94	0.09	2.14**	0.01	15.24
		G × E	4.71	94	0.05	1.15	—	—
		Error	8.3	190	0.04		0.04	53.6
	ZnZ	Environ	0	1	0	0	—	—
		Genotype	173.25	94	1.84	2.63**	0.29	20.71
		G × E	73.41	94	0.78	1.11	—	—
		Error	133.34	190	0.7		0.701	50.94
	CZnZ	Environ	0	1	0	0	—	—
		Genotype	165.15	94	1.76	2.51**	0.26	18.89
		G × E	82	94	0.87	1.25	—	—
		Error	132.85	190	0.7		0.7	50.94
Mn	None	Environ	1162.73	1	1162.73	42.41**	5.51	9.96
		Genotype	6729.55	102	65.98	2.41**	9.64	17.41
		G × E	2608.48	102	25.57	0.93	—	23.1
		Error	5647.46	206	27.41		27.41	49.53
	CMn	Environ	0.02	1	0.02	0.67	—	—
		Genotype	15.28	102	0.15	5.3**	0.03	33.74
		G × E	6.41	102	0.06	2.22**	0.03	34.89
		Error	5.82	206	0.03		0.03	31.37
	MnZ	Environ	0	1	0	0	—	—
		Genotype	226.88	102	2.22	5.03**	0.45	33.03
		G × E	93.95	102	0.92	2.08**	0.46	34.15
		Error	91.17	206	0.44		0.44	32.82
	CMnZ	Environ	0	1	0	0	—	—
		Genotype	235.41	102	2.31	6.74**	0.49	36.29
		G × E	106.03	102	1.04	3.03**	0.52	38.39
		Error	70.56	206	0.34		0.34	25.32

^a^ Environ. represents the changes in the planting fields, Genotype represents the genotypic variations in Pop6-RIL, and G × E represents the interactions between Environ. and Genotype.

^b^ Fischer test; *p < 0.05 and **p < 0.01 represent statistical significance.

^c^ The expected variance (δ^2^) in the total phenotypic variance was estimated to describe the phenotypic contribution (Con%) of each genetic component using a random model in two-way ANOVA.

Furthermore, the two normalization approaches were combined and determined through the Z-scores of comparative mineral accumulations (CZ), such as comparative grain-Cd Z-score (CCdZ), comparative grain-Mn Z-score (CMnZ), and comparative grain-Zn Z-score (CZnZ). Out of our expectation, it revealed that the genetic effects were not enhanced when the CZ evaluations was employed. The genetic contributions of CCdZ, CMnZ, and CZnZ were comparable to those of CCd, CMn, and CZn, respectively (Table 1). For instance, the genetic effects of CCd (28.85%) or CdZ (31.79%) were only slightly different from those of the CCdZ evaluations method (31.51%; Figure 1B). Hence, our results suggested that the combination of the comparative mineral accumulation approach with the Z-score is unnecessary for further boost the genetic effects, and the comparative mineral accumulation approach could be workable in boosting the genetic effects on phenotypic variation, with the exception of Zn accumulation in rice grains.

### Phenotypic variations in mineral accumulation in the rice grains of two RIL populations sharing the same *trj* rice variety

**FIGURE 2:**
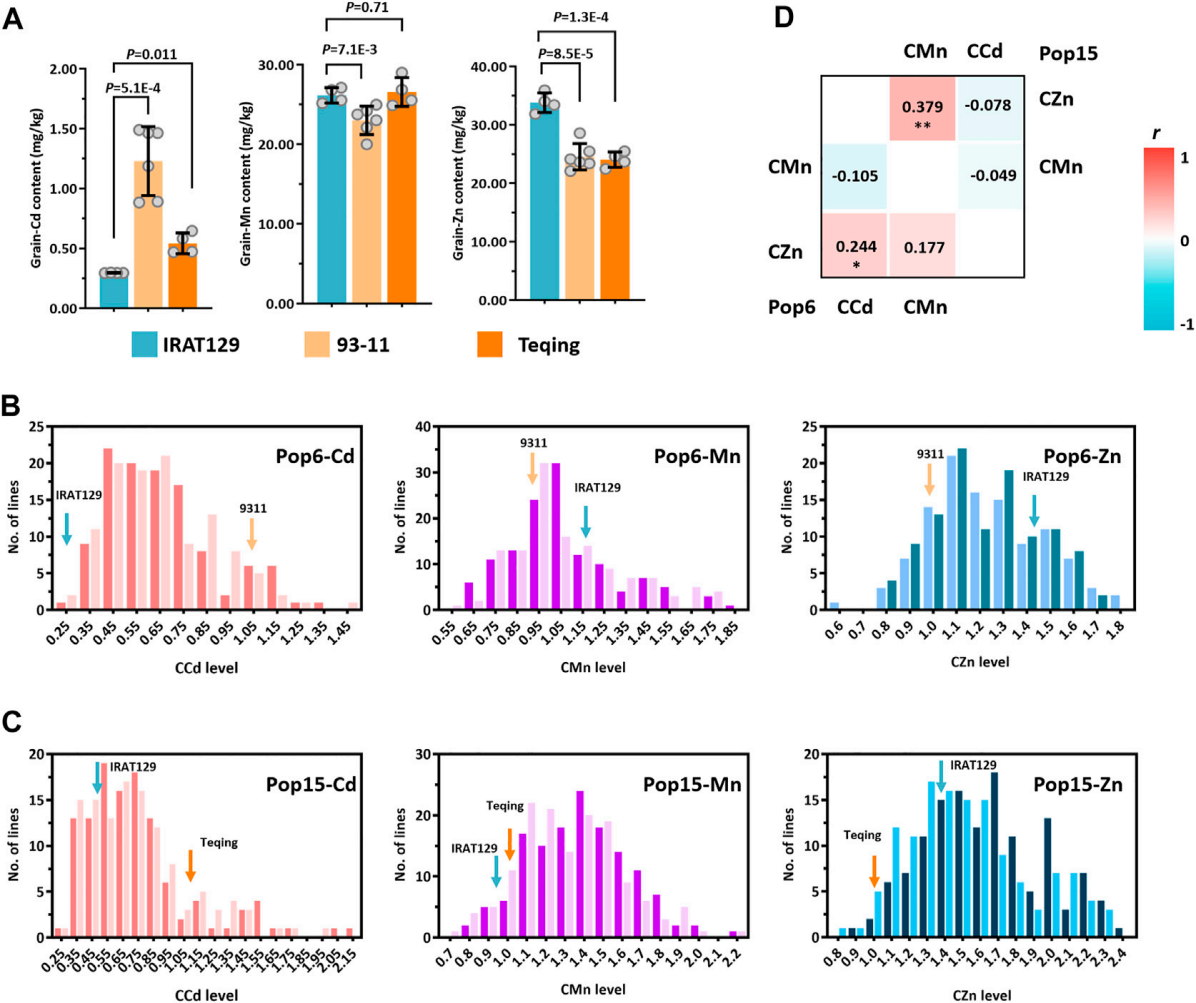
Distribution of CCd, CMn, and CZn in two RIL populations derived from “IRAT129”, “93–11”, and “Teqing” varieties. **(A)** Comparison of the accumulation of grain-Cd, grain-Mn, and grain-Zn in the grains of the three parental varieties of rice. **(B)** and **(C)** Distribution of CCd, CMn, and CZn in the two RIL populations. The arrows represent the performance of the parental lines. **(D)** The results of correlation analysis of the mineral accumulation in the two RILs. The correlation coefficients (r) are labeled, and *p < 0.05 and **p < 0.01 represent significant differences.

Significant differences in mineral accumulation were detected among the “IRAT129”, “93–11”, and “Teqing” varieties (*p* < 0.05; Figure 2A). The accumulation of Zn was higher in the grains of the “IRAT129” variety, while the accumulation of Cd was significantly lower in the “IRAT129” variety compared to that of the “93–11” or “Teqing” varieties. Additionally, the accumulation of Mn in the “93–11” variety was significantly lower than that of the other two varieties. The grain-Cd level of the “IRAT129” variety was 24.1% and 54.7% of that of the “93–11” and “Teqing” varieties, respectively. On the contrary, the grain-Zn level of the “IRAT129” variety was 1.37-fold and 1.41-fold that of the “93–11” and “Teqing” varieties, respectively. However, the grain-Mn levels were similar in the “IRAT129” and “Teqing” varieties, and were significantly higher than that of the “93–11” variety (*p* = 0.71; Figure 2A). Thus, the two RILs (Pop6-RIL and Pop15-RIL) derived from the parental varieties could be used to unveil the genetic controls driving the grain-Cd, grain-Mn, and grain-Zn accumulated differences.

The phenotypic variations were determined using the comparative mineral accumulation normalization approach, and large variations in CCd, CMn, and CZn emerged for both the RIL populations, indicating continuous segregation distribution in both populations (Figures 2B, C). Unlike CMn, the phenotypic distribution of CCd and CZn in the RILs varied from those of the parental varieties. The phenotypes displayed high levels of the coefficient of variation (CV) in both Pop6 and Pop15, which were 36.9% and 48.6%, respectively, for CCd, 24.5% and 18.6%, respectively, for CMn, and 16.4% and 19.8%, respectively, for CZn. The high CV of the phenotypes in both RIL populations suggested the presence of great genetic variations among these phenotypes, especially in the CCd levels, which had the highest CV (Figures 2B, C; Supplementary Table S1). There were slight variations in the performance of CCd, CMn, and CZn between the two planting locations, which suggested that the environmental effect was nullified by phenotypic normalization in both the populations (Supplementary Table S1).

In the meantime, the relationships among the accumulation of Cd, Mn, and Zn in the rice grains of the two populations were also investigated in this study. Through the comparative mineral accumulations in rice grains, the correlations relationships between the variations in the accumulation of Cd, Mn, and Zn in the RILs was additionally investigated and the Pearson’s correlation coefficient (*r*) was subsequently calculated (Figure 2D). There was a significantly weak positive correlation between CCd and CZn in Pop6-RIL (*r* = 0.244 < 0.3; *p* < 0.05); no significant correlations were observed between the CCd and CMn (*r* = −0.105; *p* > 0.05) or between the CMn and CZn (*r* = 0.177; *p* > 0.05). The CMn and CZn exhibited a significant moderate positive correlation in Pop15-RIL (*r* = 0.379, *p* < 0.01). And no significant correlation between CCd and CMn (*r* = −0.049; *p* > 0.05) or between CCd and CZn (*r* = −0.078; *p* > 0.05). The results indicated differences in the genetic controls driving the variations in the accumulation of the three minerals in the two populations, which agreed with the results of our previous studies (Sun et al., 2016; Tan et al., 2020a).

### The linkage map and the genetic controls driving the variations in the accumulation of Cd, Mn, and Zn in the RIL populations

After discarding the unsuitable polymorphic markers based on the results of the SSR assay, total of 142 and 123 genome-wide markers were employed to build the genetic map of Pop6-RIL and Pop15-RIL. The genetic map of Pop6-RIL had a total genetic distance of 1793.7 cM across all the 12 chromosomes, and the average distance between marker pairs was 12.2 cM. And the genetic map of Pop15-RIL covered all the 12 chromosomes, with an approximate genetic distance of 2,298 cM, and a mean distance of 18.7 cM between marker pairs (Supplementary Figure S1). Considering our two RIL populations were derived from the intersubspecies intercrosses between *indica* and *japonica* rice varieties, the total genetic distance of Pop6-RIL and Pop15-RIL were longer than those populations derived from intraspecific intercrosses, which the genetic distances were similar with the genetic maps in other previous studies (Sato et al., 2011; Zhang et al., 2014; Wang et al., 2019). In additions, based on the physical locations of the markers employed in our genetic maps (RiceVarMap, http://ricevarmap.ncpgr.cn/), the orders of makers were not found to be reversed, or malposition. No distinct gaps were created when the genetic maps were built except for two genomic regions. The genetic distances over 50.0 cM in the two genetic maps happen in two genomic locations. In Pop6-RIL, the genetic distance between markers of RM1163 and R6ID1694 on chromosome 6 was determined as 56.9 cM, and in Pop15-RIL, the genetic distance between markers of R1ID2211 and R1ID2673 on chromosome 1 was determined as 76.9 cM. These two genetic gaps might be attributed to multiple recombination events in the two regions when the two RIL populations were developed.

**FIGURE 3:**
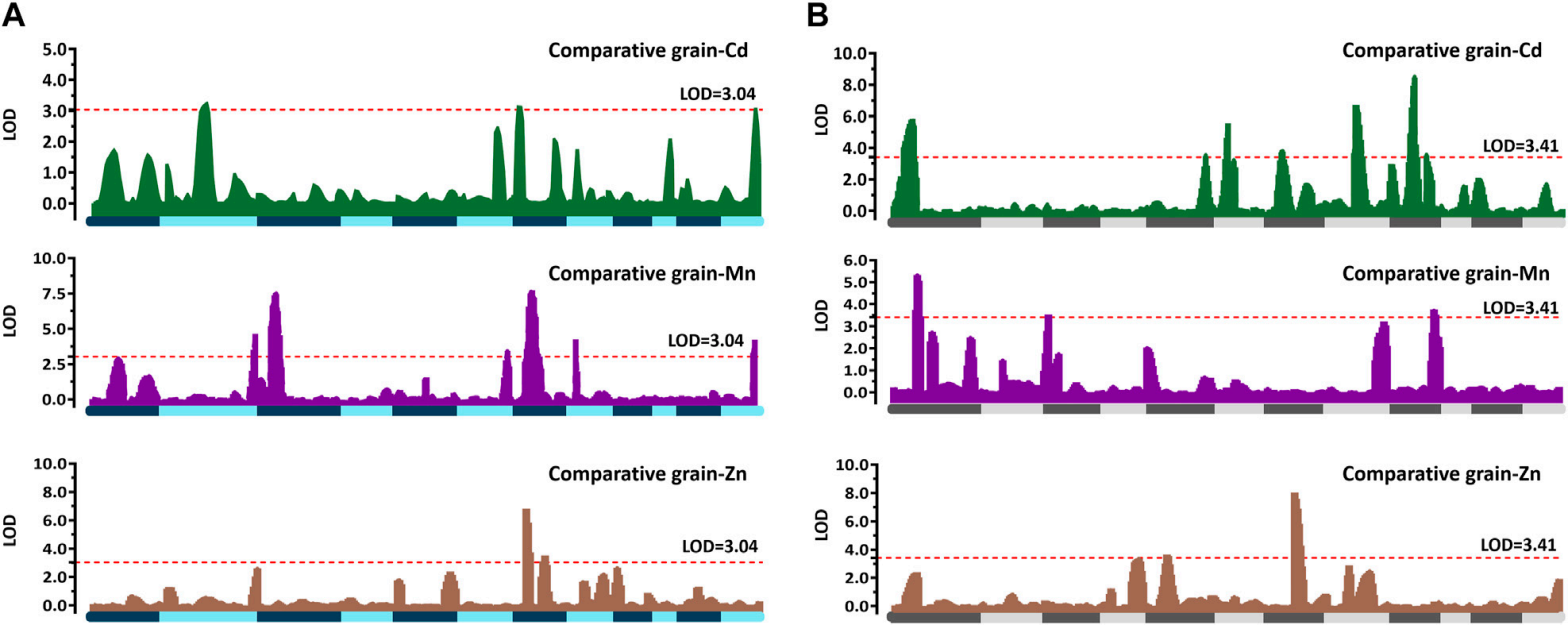
QTL identifications responsible for the variations in CCd, CMn, and CZn the two RIL populations. The horizontal axes represent the 12 chromosomes assembled in order, and all the detected QTLs are mapped to the respective positions. The regions in green, purple, and brown indicate the QTLs detected based on phenotypic variations using the comparative grain-Cd, grain-Mn, and grain-Zn levels in Pop6-RIL **(A)** and Pop15-RIL **(B)**. The dotted red lines indicate the threshold LOD score of 3.04 in Pop6-RIL, and 3.41 in Pop15-RILs determined using permutation test with 1,000 runs.

QTL mapping was performed based on the CCd, CMn, and CZn variations in the two RILs. No additive effect QTLs were detected between RM1163 and R6ID1694 on chromosome 6 in Pop6-RIL or between R1ID2211 and R1ID2673 on chromosome 1 in Pop15-RIL. For Pop6-RIL, a total of ten additive QTLs were identified which controlled the grain-Cd, grain-Mn, and grain-Zn variation (Figure 3A; Table 2). Two QTLs, namely, *qCd2*, and *qCd7*, were detected on chromosome 2 and 7, with LOD values of 3.21 and 3.11, respectively, which controlled the for grain-Cd variation. These two QTLs with negative additive effects suggested that the alleles derived from “IRAT129” could lower the grain-Cd levels and explained approximately 8.91% and 9.44%, respectively, of the phenotypic variation. A total of six QTLs were found to be associated with grain-Mn variation in the population with LOD values >3.04. Of these, two QTLs, namely, *qMn3* and *qMZ7*, had the highest ratio of phenotypic variation, which was 10.59% and 16.41% for grain-Mn variation and LOD values of 7.52 and 7.78, respectively. The alleles of “IRAT129” in the two QTLs could enhance the phenotypic variation. As regard to grain-Zn variation, two QTLs were detected being associated with variation at LOD values >3.04 (Table 2). And the *qMZ7* on chromosome 7 was found to be responsible for the grain-Mn variations (LOD = 7.78) as well as for grain-Zn variation (LOD = 3.53), and was therefore identified as a pleiotropic QTL (Figure 3A Table 2). For Pop15-RIL, a total of 12 QTLs were found to be responsible for the grain-Cd, grain-Mn, and grain-Zn variations at LOD >3.41 (Figure 3B). Total nine QTLs were found to be responsible for grain-Cd variation, preforming no more than 10% of contributions to phenotypic variations (Table 2). The QTL on chromosome 7, as *qCd7*, was detected at LOD value of 3.81 with the similar genetic location of the QTL from Pop6-RIL displaying a negative additive effect of 0.113 and explaining 4.25% of phenotypic variation. Three QTLs were found to be associated with grain-Mn variations at LOD >3.41, the QTL on chromosome 3, as *qMn3*, was detected at the similar genetic location of that being detected in Pop6-RIL, and displaying 7.01% of the comparative grain-Mn variation at LOD of 3.53 (Table 2). While for the grain-Zn variation in this population, only two QTLs were detected as *qZn5* and *qZn7*, performed a high genetic explanation for comparative grain-Zn variation at LOD values of 3.64 and 8.04. The *qZn5* allele of “IRAT129” increased the comparative grain-Zn level, and explained 11.77% of phenotypic variation. Additionally, the *qZn7* allele in ‘IRAT129’ reduce the comparative grain-Zn level, and explained 22.45% of the grain-Zn variation in Pop15-RIL (Table 2; Figure 2B).

**TABLE 2:** QTLs responsible for comparative mineral accumulation (CCd, CMn, and CZn) in the grains of the two RIL populations.

Populations	Traits	QTLs	Chr^a^	Interval	LOD^b^	PVE (%)^c^	Add^d^
Pop6-RIL IRAT129/93–11	Cd	*qCd2*	2	R2ID2035-RM1385	3.21	8.91	−0.094
		*qCd7*	7	RM6081-R7ID903	3.11	4.44	−0.065
	Mn	*qMn2*	2	R2ID3326-R2ID3533	4.72	8.02	0.078
		*qMn3*	3	R3M10-R3ID1077	7.52	10.59	0.099
		*qMn6*	6	R6ID2660-R6ID2826	3.51	4.62	−0.06
		*qMZ7*	7	R7ID1740-RM346	7.78	16.41	0.112
		*qMn8*	8	R8ID1537-R8ID1867	4.32	6.81	−0.074
		*qMn12*	12	R12ID2339-RM5479	4.29	5.69	0.066
	Zn	*qZn7*	7	R7ID1279-R7ID1506	6.83	6.19	−0.082
		*qMZ7*	7	RM346-RM6835	3.53	4.00	0.065
Pop15-RIL IRAT129/Teqing	Cd	*qCd1*	1	R1ID0894-R1ID1040	5.90	8.80	0.163
		*qCd5*	5	R5ID2432-R5M43	3.55	2.86	−0.093
		*qCd6*	6	R6ID0227-R6ID0456	5.62	5.98	−0.141
		*qCd7*	7	R7ID0431-R7ID0903	3.81	4.25	−0.113
		*qCd8*	8	R8ID1914-R8M33	6.78	6.06	−0.142
		*qCd9.1*	9	R9ID688-R9ID1363	8.53	10.67	0.193
		*qCd9.2*	9	R9M30-R9ID1738	3.57	2.97	−0.095
	Mn	*qMn1*	1	R1ID1040-R1ID1157	5.34	11.76	0.086
		*qMn3*	3	R3M10-R3ID0725	3.53	7.01	0.071
		*qMn9.2*	9	R9ID1738-R9M42	3.76	7.11	−0.071
	Zn	*qZn5*	5	R5ID0352-R5ID0553	3.64	11.77	0.159
		*qZn7*	7	R7ID1319-R7ID1717	8.04	22.45	−0.153

^a^ Chr. represents the chromosome.

^b^ The logarithm of odds (LOD) significance threshold of 3.04 in Pop6-RIL, and 3.41 in Pop15-RILs, determined using permutation test with 1,000 runs.

^c^ Phenotypic variation explained by the related QTLs.

^d^ Additive effects; the positive values indicate the alleles of the “IRAT129” variety that increase phenotypic variations, and the negative values indicate alleles of “IRAT129” that reduce phenotypic variations. In comparative accumulation levels, the unit of the additive effect is relative accumulation level compared to the control varieties, in Pop6 the control was variety “93–11”; In Pop15-RIl, the control variety was variety “Teqing”; While the in Z-score levels, the unit of the additive effect is the mathematical distance to the mean level of the whole performance of the populations.

The italic values represent the QTL loci.

In order to further confirm that the genetic contributions to phenotypic variations could not be further improved with the CZ evaluation approach, QTL detections were performed based on the CZ values for comparing the differences between the two normalization approaches. Interestingly, the QTLs leading to comparative mineral accumulated variation or CZ variation were located in nearly the same genomic regions with the linkage markers, and exhibited minor differences in the LOD scores, contribution values, or additive effects (Table 2; Supplementary Table S2). The results suggested the acceptability of our deduction regarding the genetic performance of the phenotype normalization approaches, and suggested that the genetic effects could not be further enhanced by combining comparative mineral accumulation evaluations with the Z-score evaluations to further boost the genetic effects.

### Three QTLs leading to grain-Cd, grain-Mn, and grain-Zn variation perform ecotypic divergence among rice varieties

**FIGURE 4:**
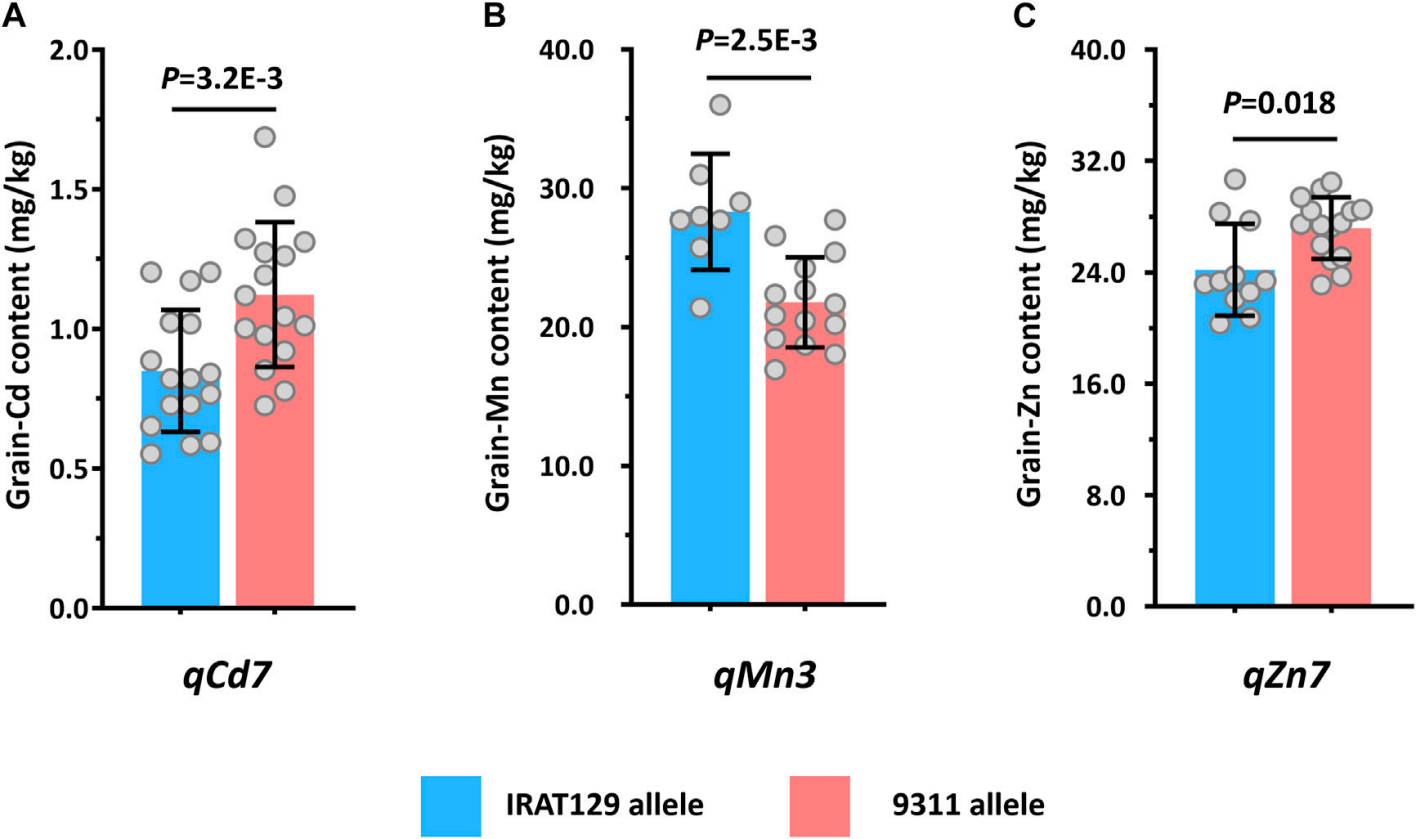
Validation of the detected QTLs by analyzing the performance of the three QTLs in different planting environments. Comparison of the performance of grain-Cd, grain-Mn, and grain-Zn in the presence of *qCd7* **(A)**, *qMn3*, **(B)**, and *qZn7* **(C)** in a paddy field contaminated with Cd.

In this study, two RILs were developed from cultivars with genetic difference between *trj* and *ind* rice varieties. The QTLs detected from them jointly revealed the potential popular genomic regions associated with the ecotypic divergence responsible for the mineral accumulation variation in rice grain. Thus, based on the genomic regions linked with markers of the detected QTLs (Figures 3A, B), three QTLs, namely, *qCd7*, *qMn3*, and *qZn7*, were characterized being associated with the same phenotypic variation for grain-Cd, grain-Mn, and grain-Zn variation in both RILs. According to performance of these QTLs, the alleles from “IRAT129” variety of the three QTLs could reduce the grain-Cd and grain-Zn performance but increase the grain-Mn levels in both the RILs (Table 2). This suggested that these alleles might be from the gene pool of *trj* rice (Figures 3A, B; Table 2). In order to determine whether the alleles of “IRAT129” in the three QTLs could affect the relevant phenotypic differences, some RIL lines harboring the homologous alleles from the “IRAT129” variety were selected based on the genotypes of Pop6-RIL lines. After planted in a Cd contaminated paddy field with soil Cd levels of 0.5–0.9 mg/kg and pH ranging from 5.4 to 6.1, differences in grain mineral accumulation in these lines harboring homologous alleles from the “IRAT129” of the QTLs were subsequently determined (Figure 4). For *qCd7*, the “IRAT129” allele could significantly lower the grain-Cd levels by approximately 24.3% (*p* = 3.2E-3; Figure 4A). For *qMn3*, the “IRAT129” allele significantly enhanced the grain-Mn levels by approximately 23.0% (*p* = 2.5E-3; Figure 4B). While for *t*he *qZn7*, the grain-Zn levels were significantly reduced approximately 11.0% at presence of “IRAT129” allele (*p* = 0.018; Figure 4C). Thus, all the results suggested three QTLs might be the characteristic QTLs derived from the genetic divergence between the *trj* and *ind* ecotypes.

**FIGURE 5:**
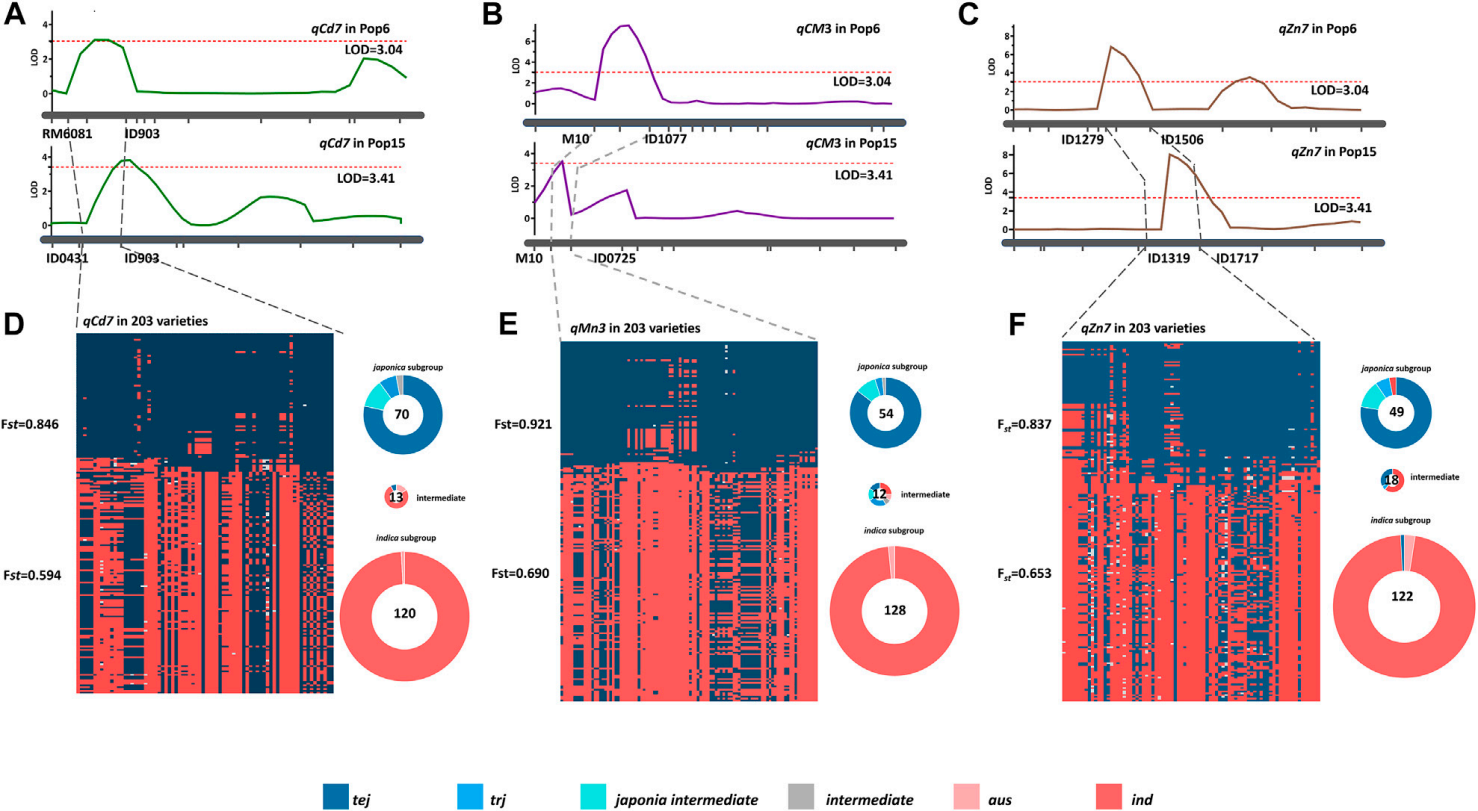
Three specific QTLs detected from *trj* variety “IRAT129” affecting the grain-Cd, grain-Mn, and grain-Zn accumulation in rice grain, and their subspecies divergence analysis. Genomic location of the QTLs **(A)** *qCd7*, **(B)** *qCM3*, and **(C)** *qZn7* determined by QTL mapping of the two RIL populations. Subspecies divergence analysis of **(D)** *qCd7*, **(E)** *qCM3*, and **(F)** *qZn7* based on the RiceVarMap 2.0 database. The red and the blue plots represent the genetic components of the *indica* and *japonica* varieties, respectively, while the numbers in the circles indicate the number of *indica* and *japonica* varieties in the collection. All the ecotypes except *aro* (*ind*, *tej*, *trj*, and *aus*) and the intermediates are indicated by different colors. The *aro* ecotype is missing from the analysis as it was not analyzed in the study.

According to the genome information, *qCd7* mapped to the 5.60–9.03 Mb genomic region on chromosome 7, *qMn3* mapped to the 6.10–10.70 Mb genomic region on chromosome 3, and *qZn7* mapped to the 13.19–17.17 Mb genomic region on chromosome 7 (Figures 5A–C). These genomic regions are located in the genomic region associated with subspecies differentiation and experiencing adaptive selection (Wei et al., 2021; Zheng et al., 2022). We therefore suspected that subspecies differentiations, especially the ecotype differentiations, in these genomic regions of the three QTLs leads to the differences in mineral accumulation in rice grain. To test our assumptions, the rice subspecies differentiations on the three QTL genomic regions of *qCd7*, *qMn3*, and *qZn7* were investigated using the RiceVarMap database (http://ricevarmap.ncpgr.cn/), which is a comprehensive database for studying the genomic variations in rice (Zhao et al., 2021). A total of 122, 115, and 134 SNPs were randomly distributed across the three genomic regions of *qCd7*, *qMn3*, and *qZn7*, respectively, were captured from 203 rice-variety collection from the database, displaying about 30 kb per SNP covering the QTL regions. Based on the nucleotide polymorphisms of these SNPs, two group were clustered using STRUCTURE (K = 2) in each QTL genomic region (Figures 5D–F). Distinct genetic divergence between the subgroups were shown, which the *F*_st_ values of each group were determined more than 0.5. The *F*_st_ values of the two SNP clusters in each of the genomic regions of *qCd7*, *qMn3*, and *qZn7* were 0.846 and 0.594 (Figure 5D), For *qMn3*, the *F*_st_ values of two groups were 0.921 and 0.690 (Figure 5E), and for *qZn7*, the *F*_st_ values of two groups were 0.837 and 0.653 (Figure 5F). According to the information obtained from the 203 rice-variety collection, the two groups could be defined as the *indica* and *japonica* subgroups. For *qCd7*, 70 (∼34.5% of the rice variety) and 122 (∼60% of the rice variety) varieties were clustered in the *japonica* and *indica* groups, respectively (Figure 5D). For *qMn3*, approximately 31.0% and 63.0% of the rice variety were assemble to the *japonica* group and *indica* group (Figure 5E). For *qZn7*, ∼34.4% and 59.1% of the varieties were clustered in the *japonica* and *indica* groups, respectively (Figure 5F). None of the *trj* ecotype varieties were clustered in the *indica* group based on the nucleotide polymorphisms in the genomic regions of the three QTLs, irrespective of the groups (Figures 5D–F). Taking together, the results of rice subspecies analysis provided supporting further information to our assumption. The results of genomic variation analysis revealed that allelic variations in *qCd7*, *qMn3*, and *qZn7* were prevalent among the different rice ecotypes and that subspecies differentiation could be the driving force for these allelic variations, which led to the mineral accumulation.

## Discussion

There are considerable phenotypic variations in mineral accumulations in rice grains among different varieties (Zhang et al., 2014), which poses potentials for genetic improvements on the mineral accumulated performance *via* marker-assisted selection (MAS). Studies in the past few decades have revealed the genetic patterns that shape the diversity among different varieties of rice, and the variations in mineral accumulation in rice grains are typical quantitative traits that are largely determined by environmental factors, such as water regime and soil chemical properties (Zhang et al., 2014; Sun et al., 2016; Tan et al., 2020a). Evidences that the genetic controls driving mineral accumulation in rice grains are quite high (*h* ^2^ = 0.39∼0.87; Yang et al., 2018; Tan et al., 2020a) has been conferred; however, multiple different phenotypic performance from one genotype were often found (Tan et al., 2020b; Sun et al., 2022). Consequently, this leads to an ever-changing phenotypic performance of the mineral accumulation in rice grain among rice varieties/lines, which poses challenges during the exploration of genetic controls towards the further genetic improvement in rice. In order to address the issue of phenotypic analysis, different studies on natural variations in mineral accumulation in rice grains were carried out through normalizing the phenotypic variations, such as calculating the best linear unbiased prediction (BLUP) values, determining the Z-score values, or evaluating the comparative mineral accumulation values (Wang et al., 2019; Tan et al., 2020a; Tan et al., 2020b; Sun et al., 2022). In this study, we extended our previously developed CCd evaluation approach to determine other minerals accumulations in rice grains, including Mn and Zn. We additionally investigated the performance of different phenotype normalization approaches for phenotypic investigations in a genetic population. No matter which approach was adopted for estimating the grain-Cd, grain-Mn, and grain-Zn variation, including the comparative mineral accumulation evaluations, Z-scores evaluations, and CZ evaluations, the environmental changes did not affect the performance of the mineral accumulation variation any more in the population (Table 1; Figure 1; Supplementary Figure S2). However, the genetic contributions to phenotypic variations were not enhanced irrespective of the approach used for normalize grain-Zn variation, and the genetic contributions to the levels of grain-Zn, CZn, and CZnZ were 15.05%, 15.24%, and 18.89%, respectively (Table 1). This finding suggested that the normalization approach was incapable of enhancing the genetic contributions to grain-Zn variations.

The normalization approaches used in this study were established based on different considerations. As a powerful statistics algorithm, the Z-score determines were established to investigate the relationship between one test line and the mean performance of the population. The accuracy of this normalizing approach largely relies on the performance of the obtained data that is used as input (McLeod, 2019). On the other hand, the comparative mineral accumulation approach was established by normalizing the genetic performance of different rice varieties/lines using one genotype from the control line (Sun et al., 2022), which was based on the field-design experiment exceling in minimizing the effects of soil heterogeneity (Lin and Poushinsky, 1983). Our previous study confirmed that the CCd evaluation approach excelled in detecting stable performance of grain-Cd between different genotypes (Sun et al., 2022). Thus, taking them together, we therefore attempted to find out whether the comparative mineral accumulation approach could be further improved by calculating the CZ values, determined by combining the two aforementioned normalization approaches. Nevertheless, when synthesizing the two approaches using CZ evaluations, the genetic effects on determining the phenotypic variation was not improved further when the two approaches were combined for calculating the CZ values (Table 1; Figure 1B). The performance of the CCdZ approach was comparable to that of the CCd evaluation approach (Figure 1B), which also emerges in either grain-Mn or grain-Zn variations (Table 1). The findings suggested that the genetic contributions were not markedly enhanced by combining the two approaches, and QTL detection using different phenotype normalization approaches further confirmed this observation. The QTLs detected using the comparative mineral accumulation approach were similar to those detected by the Z-score method, and this was especially true for the QTLs associated with the variations in grain-Cd and grain-Mn. The QTLs detected based on the CZ values were identical to those detected using the comparative mineral accumulation approach; however, there were minor differences in the LOD scores, PVE%, and additive effects (Table 2; Supplementary Table S2). Taking together, the results from QTL detections suggested that the comparative mineral accumulation approach excelled in exploring the genotypic differences responsible for the stable phenotypic variations, especially in determining the grain-Cd and grain-Mn variation in rice. However, the approach had little effect on improving the normalization approach for comparative evaluation of mineral accumulation in different varieties/lines of rice when combined with the Z-score method.

It is generally accepted that significant differences on mineral accumulation in rice grain are prevalent between rice subspecies or rice ecotypes (Uraguchi and Fujiwara, 2013; Sun et al., 2016; Tan et al., 2020a). Evidences that the *indica* rice often accumulated much more Cd but less Mn and Zn than did *japonica* rice have been proposed (Grant et al., 2008). Of the five rice ecotypes (Garris et al., 2005), the *tej* and *trj* ecotypes, belonging to the *japonica* variety, often accumulate higher levels of Mn and Zn but lower levels Cd in grain than did the *aus* and *ind* rice (Sun et al., 2016; Tan et al., 2020a). Hence, full explorations the genotypic variation in rice ecotypes could benefit to biofortification at increasing the grain-Zn and grain-Mn accumulation and limiting the Cd accumulation in rice grain. In this study, through QTL analysis on two RIL populations characterized by sharing one common *trj* rice variety ‘IRAT129’, the genetic controls for grain-Cd, grain-Mn, and grain-Zn were well identified (Figure 3). Comparisons of the results of QTL mapping in both the RILs led to the identification of three QTLs, namely, *qCd7*, *qMn3*, and *qZn7*, that were associated with the variations in grain-Cd, grain-Mn, and grain-Zn variation, respectively, in both the populations (Table 2; Figure 3). After verifications of the three QTLs under a contaminated paddy field, it revealed that the allele from “IRAT129” in *qCd7*, *qMn3*, and *qZn7* could reduce grain-Cd and grain-Zn levels, increase the grain-Mn levels, correspondingly (Figure 4). Subsequent SNP analysis of the genomic regions of the QTLs revealed distinct rice ecotypic divergence attributed from subspecies differentiation in rice (Figure 5). The finding agreed with the results of our previous study which reported that the allelic divergence between subspecies shapes the differences in mineral accumulation between different subspecies of rice (Sun et al., 2016; Tan et al., 2020a). Thus, after the further QTGs isolations of *qCd7*, *qMn3*, and *qZn7*, the genetic improvements on enriched mineral nutrients and/or lowered Cd accumulation in rice grain could be facilitated.

In this study, three QTLs, namely, *qCd7*, *qMn3*, and *qZn7*, responsible for the grain-Cd, grain-Mn, and grain-Zn variations, respectively, were identified based on the genetic variations between the *trj* and *ind* rice ecotypes (Figures 3, 4; Table 2). Compared to other previous studies, the three QTLs could be detected multiple times (Norton et al., 2010; Hu et al., 2016; Descalsota-Empleo et al., 2019; Perera I et al., 2019; Tan et al., 2020a; Ruang-areerate et al., 2021). However, up to now, quite few reports were known for isolating and cloning the candidate QTL genes for the three QTLs*.* Nevertheless, informative clues could still be provided for speculating candidates of *qCd7* and *qMn3*, except for *qZn7*. Three Cd-associated genes, as *OsNramp5*, *OsNramp1* and *OsHMA3*, were present in the genomic region of *qCd7* (Figure 5A). In details, *osNramp5* encodes a major transporter that responsible for mediating Cd uptake in rice root. The loss of function of the osNramp5 in *osnramp5* mutants results in extremely low levels of Cd in grains (Sasaki et al., 2012). *OsNramp1* encodes a transporter protein that responsible to Cd uptake and transport, and is expressed in the roots and leaves of rice. Differences in the expression levels of *OsNramp1* between different varieties of rice results in differences in the levels of Cd in the shoots (Takahashi et al., 2011; Zhou et al., 2017). It has been confirmed that natural variations in *OsHMA3* are responsible for differences in the sequestration of Cd in the vacuoles, which leads to variations in grain-Cd levels in rice (Ueno et al., 2010; Sui et al., 2019). About *qMn3*, two genes, namely *OsNramp2* and *MTP8.1*, were found present in the related genomic region, and could be the candidate QTGs of *qMn3*. Despite that molecular function of *OsNramp2* in rice has not been identified to date, the loss of function of *AtNramp2*, an orthologous gene of *OsNramp2* that mediates the influx of Mn in yeast, leads to the increased retention and high levels of Mn in the roots of rice plants (Gao et al., 2018). The *OsMTP8.1* gene is orthologous to *AtMTP8*, and has been found to mediate Mn homoeostasis by sequestering excess Mn into vacuoles (Chen et al., 2013). Interestingly, the different expressions of these candidate genes were also found between different lines harboring the grain-Mn diversity (Ruang-Areerate et al., 2021). Thus, after determining the allelic variations in these candidate genes between the parental varieties from different ecotypes, the potential QTG of the three QTLs could be figured out. Accordingly, the studying results obtained herein could broaden our understanding on grain-Cd, grain-Mn, or grain-Zn accumulation in rice, and the functional alleles identified from the QTLs could be employed for the genetic improvement of rice *via* the biofortification of essential mineral nutrients and limiting the accumulation of toxic elements in rice grains.

## Data Availability

Publicly available datasets were analyzed in this study. This data can be found here: http://ricevarmap.ncpgr.cn/.
