# Supplementary material for: Identification of subspecies-divergent genetic loci responsible for mineral accumulation in rice grains
Source: Front Genet. 2023 Feb 7;14:1133600. doi: 10.3389/fgene.2023.1133600 (PMC9941327; doi:10.3389/fgene.2023.1133600)
Supplement: Supplementary file 1 [file Table1.DOCX]

**Legends for Supplemental Tables and Figures**


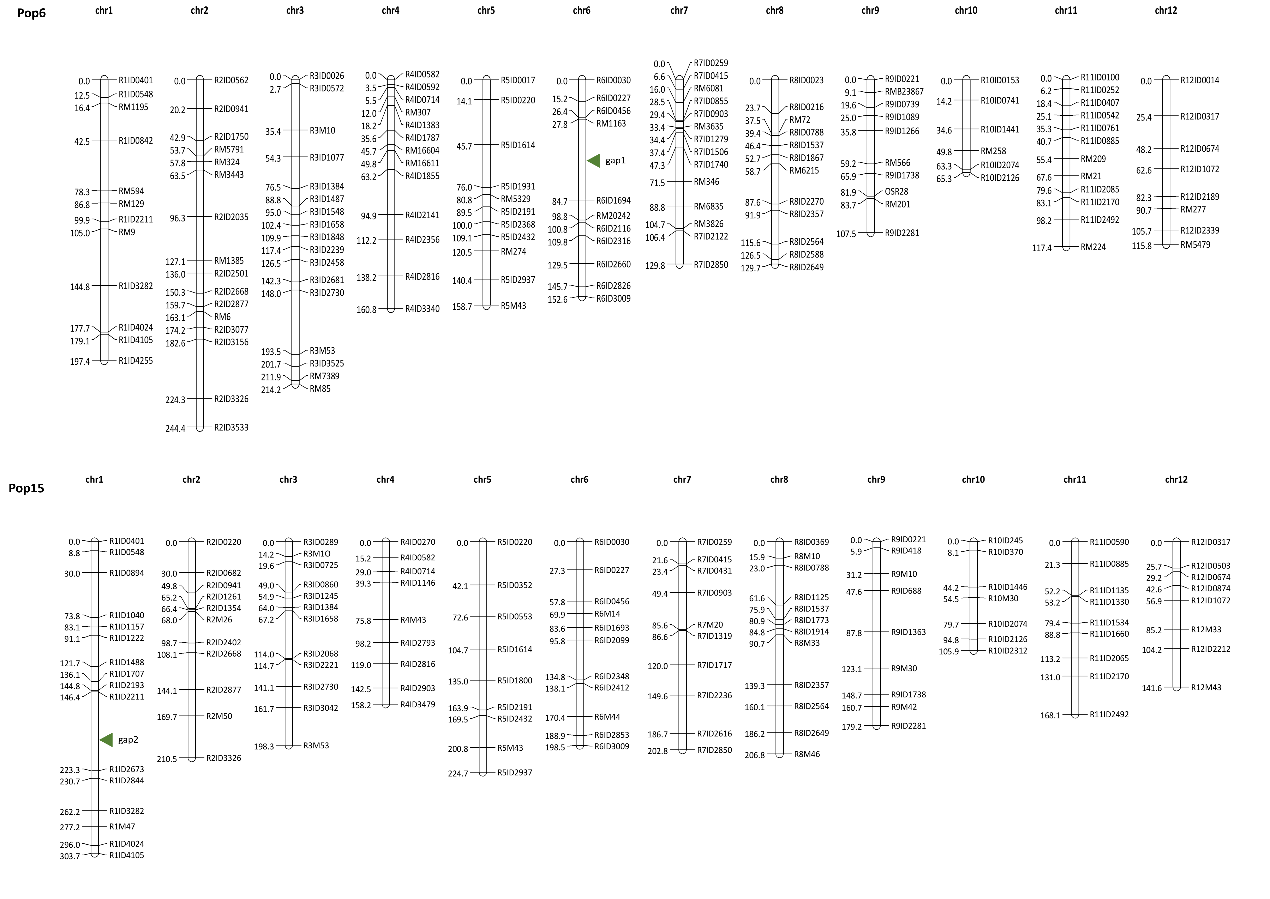


**Supplemental Figure 1 the genetic maps for the two RILs** the genetic maps were plotted using MapChart 2.3.2 software. The genetic distances of all the markers were labeled. The two triangular arrows represent two gaps in Pop6-RIL and pop15-RIL, respectively, due to their big genetic distances over 50.0 cM.


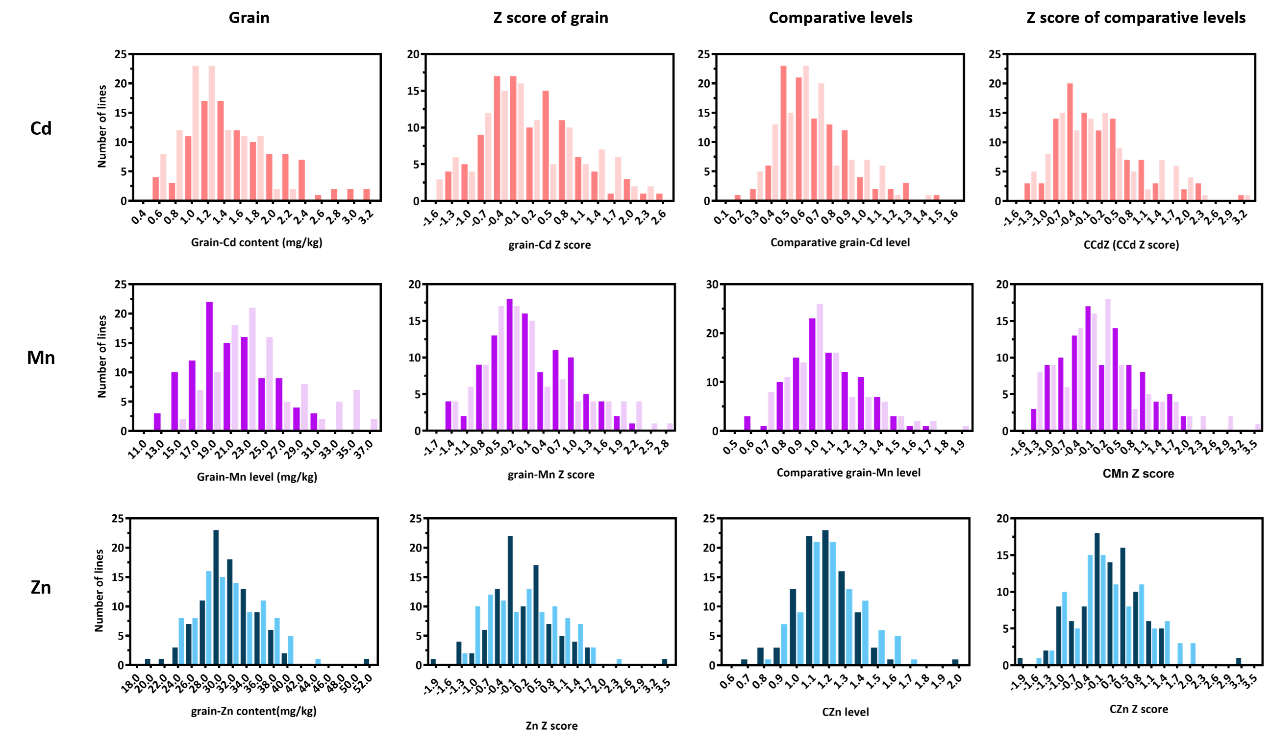


**Supplemental figure 2 the phenotypic performance of the grain-Cd, grain-Mn, and grain-Zn accumulation in rice under different normalization approaches.**

**Supplemental Table 1 the mineral accumulation performance of the two RIL population using the phenotype normalization**

|  | Traits | Repeat | range | Mean SD | C.V. (%) |
| --- | --- | --- | --- | --- | --- |
| Pop6-RIL | CCd | R1 | 0.22~1.27 | 0.61±0.23 | 37.7 |
|  |  | R2 | 0.19~1.44 | 0.60±0.23 | 38.6 |
|  |  | Mean | 0.23~1.35 | 0.61±0.22 | 36.9 |
|  | CMn | R1 | 0.55~1.81 | 1.02±0.24 | 24.4 |
|  |  | R2 | 0.47~1.71 | 1.04±0.27 | 26.2 |
|  |  | Mean | 0.55~1.75 | 1.03±0.26 | 24.5 |
|  | CZn | R1 | 0.56~1.73 | 1.18±0.24 | 21.1 |
|  |  | R2 | 0.71~1.70 | 1.17±0.22 | 19.1 |
|  |  | Mean | 0.78~1.62 | 1.18±0.19 | 16.4 |
| Pop15-RIL | CCd | R1 | 0.18~2.14 | 0.71±0.37 | 52.0 |
|  |  | R2 | 0.17~1.90 | 0.69±0.36 | 48.5 |
|  |  | Mean | 0.18~1.89 | 0.70±0.34 | 48.6 |
|  | CMn | R1 | 0.78~2.14 | 1.33±0.26 | 19.4 |
|  |  | R2 | 0.69~2.15 | 1.27±0.28 | 21.9 |
|  |  | Mean | 0.77~2.04 | 1.30±0.24 | 18.6 |
|  | CZn | R1 | 0.89~2.32 | 1.58±0.33 | 20.9 |
|  |  | R2 | 0.70~2.27 | 1.46±0.34 | 23.4 |
|  |  | Mean | 0.91~2.26 | 1.52±2.26 | 19.8 |

**Supplemental Table 2 QTLs for comparative mineral accumulation levels (CCd, CMn, and CZn), Z-score of grain accumulation levels (CdZ, MnZ, and ZnZ), and CZ values (CCdZ, CMnZ, and CZnZ) and in rice grain from the two RIL populations**

| Population | Traits | QTLs | Chr.^a^ | Interval | Comparative accumulation level | | | Z-score of grain accumulation level | | | Z-score of comparative mineral accumulation | | |
| --- | --- | --- | --- | --- | --- | --- | --- | --- | --- | --- | --- | --- | --- |
|  |  |  |  |  | LOD^b^ | PVE(%)^c^ | Add^d^ | LOD^b^ | PVE(%)^c^ | Add^d^ | LOD^b^ | PVE(%)^c^ | Add^d^ |
| Pop6-RIL  IRAT129/93-11 | Cd | *qCd2* | 2 | R2ID2035-RM1385 | 3.21 | 8.91 | -0.094 | 5.15 | 8.2 | -0.422 | 3.22 | 8.92 | -0.407 |
|  |  | *qCd6* | 6 | R6ID2316-R6ID2660 |  |  |  | 3.48 | 4.07 | 0.296 |  |  |  |
|  |  | *qCd7* | 7 | RM6081-R7ID903 | 3.11 | 4.44 | -0.065 | 3.13 | 3.58 | -0.276 | 3.12 | 4.46 | -0.282 |
|  | Mn | *qMn1* | 1 | R1ID0842-RM594 |  |  |  | 4.8 | 10.37 | -0.396 |  |  |  |
|  |  | *qMn2* | 2 | R2ID3326-R2ID3533 | 4.72 | 8.02 | 0.078 | 5.11 | 6.52 | 0.314 | 4.68 | 7.98 | 0.304 |
|  |  | *qMn3* | 3 | R3M10-R3ID1077 | 7.52 | 10.59 | 0.099 | 4.84 | 4.93 | 0.302 | 7.5 | 10.57 | 0.386 |
|  |  | *qMn6* | 6 | R6ID2660-R6ID2826 | 3.51 | 4.62 | -0.060 | 3.74 | 3.76 | -0.242 | 3.49 | 4.61 | -0.234 |
|  |  | *qMZ7* | 7 | R7ID1740-RM346 | 7.78 | 16.41 | 0.112 | 8.62 | 12.81 | 0.441 | 7.76 | 16.36 | 0.439 |
|  |  | *qMn8* | 8 | R8ID1537-R8ID1867 | 4.32 | 6.81 | -0.074 | 3.13 | 3.86 | -0.248 | 4.32 | 6.83 | -0.289 |
|  |  | *qMn12* | 12 | R12ID2339-RM5479 | 4.29 | 5.69 | 0.066 | 5.96 | 6.28 | 0.311 | 4.32 | 5.74 | 0.261 |
|  | Zn | *qZn7* | 7 | R7ID1279-R7ID1506 | 6.83 | 6.19 | -0.082 |  |  |  | 6.86 | 6.20 | -0.376 |
|  |  | *qMZ7* | 7 | RM346-RM6835 | 3.53 | 4.00 | 0.065 |  |  |  | 3.56 | 4.02 | 0.300 |
|  |  | *qZn9* | 9 | R9ID0739-R9ID1089 |  |  |  | 3.37 | 6.58 | -0.289 |  |  |  |
| Pop15-RIL  IRAT129/Teqing | Cd | *qCd1* | 1 | R1ID0894-R1ID1040 | 5.9 | 8.8 | 0.163 |  |  |  | 5.92 | 8.55 | 0.447 |
|  |  | *qCd5* | 5 | R5ID2432-R5M43 | 3.55 | 2.86 | -0.093 |  |  |  | 3.53 | 2.77 | -0.254 |
|  |  | *qCd6* | 6 | R6ID0227-R6ID0456 | 5.62 | 5.98 | -0.141 |  |  |  | 5.59 | 5.78 | -0.387 |
|  |  | *qCd7* | 7 | R7ID0431-R7ID0903 | 3.81 | 4.25 | -0.113 |  |  |  | 3.93 | 4.27 | -0.315 |
|  |  | *qCd8* | 8 | R8ID1914-R8M33 | 6.78 | 6.06 | -0.142 | 5.21 | 4.68 | -0.405 | 6.72 | 7.79 | -0.44 |
|  |  | *qCd9.1* | 9 | R9ID688-R9ID1363 | 8.53 | 10.67 | 0.193 | 3.93 | 5.56 | 0.456 | 8.54 | 10.38 | 0.531 |
|  |  | *qCd9.2* | 9 | R9M30-R9ID1738 | 3.57 | 2.97 | -0.095 |  |  |  | 3.57 | 2.88 | -0.261 |
|  | Mn | *qMn1* | 1 | R1ID1040-R1ID1157 | 5.34 | 11.76 | 0.086 |  |  |  | 5.33 | 11.77 | 0.318 |
|  |  | *qMn2.1* | 2 | R2ID2668-R2ID2877 |  |  |  | 4.84 | 8.96 | 0.412 |  |  |  |
|  |  | *qMn2.2* | 2 | R2ID2877-R2M50 |  |  |  | 4.51 | 6.7 | 0.356 |  |  |  |
|  |  | *qMn3* | 3 | R3M1O-R3ID0725 | 3.53 | 7.01 | 0.071 |  |  |  | 3.54 | 7.03 | 0.264 |
|  |  | *qMn9.1* | 9 | R9ID418-R9M10 |  |  |  | 4.42 | 5.94 | 0.346 |  |  |  |
|  |  | *qMn9.2* | 9 | R9ID1738-R9M42 | 3.76 | 7.11 | -0.071 |  |  |  | 3.77 | 7.13 | -0.264 |
|  | Zn | *qZn5* | 5 | R5ID0352-R5ID0553 | 3.64 | 11.77 | 0.159 | 5.33 | 16.62 | 0.558 | 3.66 | 11.83 | 0.488 |
|  |  | *qZn7* | 7 | R7ID1319-R7ID1717 | 8.04 | 22.45 | -0.153 | 8.01 | 23.03 | -0.442 | 8.04 | 22.41 | -0.466 |

^a^ Chr. represents the chromosome; ^b^ the logarithm of odds (LOD) significance threshold of 3.04 in Pop6-RIL, and 3.41 in Pop15-RILs determined using permutation test with 1,000 permutations; ^c^ phenotypic variation explained by the related QTLs. ^d^ Additive effects; the positive values imply the allele from “IRAT129” could increase the phenotypic variation, and the negative values suggest the allele from “IRAT129” could reduce the phenotypic variation. In comparative mineral accumulation levels, the unit of the additive effect is relative accumulation level compared to the control varieties, in Pop6 the control was variety ‘93-11’; In Pop15-RIl, the control variety was variety ‘Teqing’; While the in Z-score levels, the unit of the additive effect is the mathematical distance to the mean level of the whole performance of the populations.
